# Improving Continuity of Care of Patients Transferred Between Medical and Psychiatry Wards During the COVID-19 Pandemic and the Increasing Demands on Core Trainees to Manage Medical CoMorbidity

**DOI:** 10.1192/bjo.2022.277

**Published:** 2022-06-20

**Authors:** Paula Beaumont

**Affiliations:** NHS Forth Valley, Larbert, United Kingdom

## Abstract

**Aims:**

An existing transfer document at FVRH recognised that patients presenting to one specialty may require transfer to another depending on the changing needs of that patient. This document was not often used prior to the COVID-19 pandemic however demands for medical beds resulting in prompt return of patients to psychiatry highlighted the need to adhere to a safe transfer process. Unlike many psychiatry units where physically unwell patients are taken to ED, the MHU for Forth Valley is attached to the general hospital. This results in the view that physically unwell patients can be managed for longer before requiring transfer. Despite the proximity to the medical wards however, the unit is not equipped to manage physically deteriorating patients. This QI project aimed to improve communication between psychiatry and medical staff to improve patient safety.

**Methods:**

Patients transferred during their admission between the MHU and medical or surgical wards in May 2020, or in Oct-Dec 2021 were identified from 5 psychiatry wards. Electronic and paper notes were checked for a transfer form for each stage of transfer. Medications prior to transfer, on return and changes during admission were cross checked on Hepma, ECS and care partner as well as within documentation from medicine/surgery.

**Results:**

In May 2020, no patients admitted from medical wards had a transfer form completed, 62.5% transferred to medicine and 57.1% returned from medical wards had forms. 20% of transfers had medication errors Identified. After making the transfer form electronic and following hospital wide changes to the Trakcare and Hepma systems, 27.8% of patients admitted from medical wards had forms, 75.9% transferred to medicine and 72% of those returned from medicine had forms. There were no further medication errors identified. During the timeframes studied only 1 patient was transferred due to COVID-19 but 29 transfers were carried out for other acute physical issues.

**Conclusion:**

Changing the documentation process to make it as easy as possible for psychiatry juniors to document treatment plans for transferred patients improved continuity of care and decreased medication errors. This also ensured that patients were medically fit to return to psychiatry wards. The range of physical comorbidities that psychiatry trainees were expected to manage extends beyond caring for patients who contract COVID -19.

